# Increased Risk for Carbapenem-Resistant *Enterobacteriaceae* Colonization in Intensive Care Units after Hospitalization in Emergency Department

**DOI:** 10.3201/eid2606.190965

**Published:** 2020-06

**Authors:** Matias Chiarastelli Salomão, Maristela Pinheiro Freire, Icaro Boszczowski, Sueli F. Raymundo, Ana Rubia Guedes, Anna S. Levin

**Affiliations:** Faculdade de Medicina da Universidade de São Paulo, São Paulo, Brazil

**Keywords:** emergency department, intensive care unit, carbapenem-resistant *Enterobacteriaceae*, CRE, carbapenem resistance, hospital infection, infection control, colonization, antimicrobial resistance, bacteria, ICU, Brazil

## Abstract

Carbapenem-resistant *Enterobacteriaceae* (CRE) colonization is common in hospital patients admitted to intensive care units (ICU) from the emergency department. We evaluated the effect of previous hospitalization in the emergency department on CRE colonization at ICU admission. Our case–control study included 103 cases and 201 controls; cases were patients colonized by CRE at admission to ICU and controls were patients admitted to ICU and not colonized. Risk factors were emergency department stay, use of carbapenem, Simplified Acute Physiology Score, upper digestive endoscopy, and transfer from another hospital. We found that ED stay before ICU admission was associated with CRE colonization at admission to the ICU. Our findings indicate that addressing infection control problems in EDs will help to control carbapenem resistance in ICUs.

*Klebsiella pneumoniae* carbapenemase (KPC), described in 1996, is an enzyme capable of hydrolyzing all β-lactam antimicrobial drugs known at the time (*1*). Since then, other carbapenemases have been described in *Enterobacteriaceae* all over the world, leading to a substantial increase in resistance to antimicrobial drugs (*2**,**3*).

Surveillance data from central line–associated bloodstream infections (CLABSI) in intensive care units (ICUs) in the state of São Paulo, Brazil, demonstrated an increase of carbapenem-resistant *K. pneumoniae*, from 14% in 2011 to 55% in 2017 (*4*). In 2017, *K. pneumoniae* was the most frequent species causing CLABSI (20%) in São Paulo.

Hospital das Clínicas of the University of São Paulo has routinely performed CRE screening for patients admitted to ICU since January 2014. Early identification and isolation of colonized patients was implemented to decrease secondary colonization. Concomitant training sessions for hand hygiene and contact precautions took place during this period. Despite all efforts, ICUs had a high colonization pressure (17% –29%, mean 21%) due to admission of colonized patients, mainly from EDs (I. Boszczowski, unpub. data).

In 2016, we found that 7% of patients admitted to the ED were positive for CRE. However, among those who were negative at admission, 18% became colonized during their stay in the ED. These findings led us to hypothesize that hospitalization in the ED may be a risk factor for CRE colonization in other units of the hospital (*5*); ≈60% of the patients admitted to ICUs come from hospitalizations in the ED. We evaluated the effect of hospitalization in the ED on CRE colonization at the time of admission to an ICU.

## Methods

### Setting

Hospital das Clinicas is a 2,200-bed public tertiary-care hospital in São Paulo and is the largest hospital complex in Latin America. The main building has ≈1,000 beds and is the location of the ED and most of the hospital’s ICU beds (10 ICUs and 109 intensive care beds).

**Figure 1:**
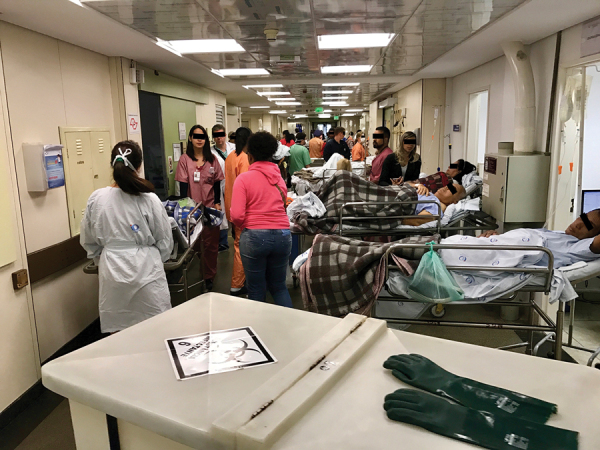
A corridor in the emergency department of Hospital das Clínicas, São Paulo, Brazil, showing patients on stretchers, December 2016.

The ED is a very busy unit. In 2018, 69,000 emergency consultations were performed. The average hospitalization rate in the ED is 150 patients/week, and median length of stay is 6 days. The ED has 50 beds for hospitalization, but occupancy often exceeds 90 beds, with patients on stretchers and often in corridors (Figure 1).

Approximately 60% of ICU patients are admitted from the ED. To monitor and control CRE colonization, CRE surveillance cultures are performed on all patients admitted to ICUs at the time of admission and placed under contact precautions until the return of results. Colonized patients with CRE remain under contact precautions for their entire stay in the unit.

### Microbiology

Surveillance cultures are performed at the clinical microbiology laboratory in accordance with the institution’s standard methodology. Rectal swab specimens from patients are incubated overnight in thioglycolate broth. Positive growth samples are plated on MacConkey agar with ertapenem, imipenem, and meropenem discs. If there are colonies suggestive of *Enterobacteriaceae* growth within the carbapenems’ disk halo, these colonies are isolated and identified by matrix-assisted laser desorption/ionization time-of-flight mass spectrometry, as recommended by Clinical and Laboratory Standards Institute (*6*).

### Study Design

We conducted a retrospective case–control study with patients hospitalized in ICUs at HC during September 2015–July 2017. This study used 2 controls for each case. We obtained cases from the infection control department database, which compiles all cases of positive surveillance cultures. Patients who were hospitalized >1 time in ICUs were considered only once, during their first hospitalization.

We defined a case as a patient admitted to one of the ICUs during 2015–2017 who had a positive CRE surveillance culture collected within 2 days of admission. We defined a control as a patient admitted to the ICU whose surveillance cultures collected within the first 2 days of admission were negative. Colonization or prior infection with CRE reported at admission were excluding criteria. We paired controls by ICU and hospitalization period, with a maximum interval of 1 week from the admission of the cases. When >2 patients were eligible as controls for a case, we randomly chose 2 from all the potential controls. The proportion of controls admitted in the ICUs from the ED was similar to the proportion of patients coming from the ED found in our historical series. CRE screening methodologies were the same for all patients in the study period, whether they were cases or controls.

We collected data from medical records for demographic variables, hospitalization records before ICU admission, clinical characteristics at time of ICU admission, severity scores and organ failures, indwelling devices, clinical procedures before ICU admission, concurrent conditions, use of antimicrobial drugs (for ≥48 hours before ICU admission) and infection before ICU admission, previous colonization, infection by CRE, length of hospital stay, and death. We defined CLABSI according to the 2018 U.S. Centers for Disease Control and Prevention definition (*7*).

We used REDCap (Research Electronic Data Capture) program (*8*) to create a data collection tool and database. The Ethics and Research Committee of Hospital das Clinicas da Faculdade de Medicina da Universidade de Sao Paulo approved this study (number CAAE: 91604518.9.0000.0068).

### Statistical Analysis

We calculated sample size and determined a minimum requirement of 99 cases and 198 controls for 80% power. We assumed that 35% of the cases had an ED stay >2 days. We performed statistical analysis using Stata version 16 (StataCorp, https://www.stata.com) and SPSS Statistics 11.5 (http://www.ibm.com). We compared cases with controls using the Wilcoxon or McNemar test when appropriate. All tests were 2-tailed, with 95% CIs; we considered p < 0.05 statistically significant. For variables with p < 0.05 in the bivariate analysis, we conducted multivariate analysis with other confounding variables in a conditional logistic regression model. Length of ED stay was a continuous variable and was transformed into a dichotomic variable using SPSS decision tree tool, and for the final model we chose the one with a better fit. We used stepwise backward modeling for the conditional logistic regression and kept the most significant variables in the final model. We used 2 models, one using length of ED stay as a continuous variable and the other as a dichotomous variable. Smoking and sepsis variables comprised more than 40% of missing data (Tables 1 and 2) and were dropped out.

**Table 1:** Characteristics of patients, bivariate analysis, and conditional logistic regression of variables potentially associated with colonization by carbapenem-resistant *Enterobacteriaceae* at ICU admission, Hospital das Clínicas, São Paulo, Brazil, September 2015–July 2017*

Covariate	Bivariate analysis					Conditional logistic regression	
	Cases	Controls	OR (95% CI)	p value		OR (95% CI)	p value
Female sex	34/103 (33)	91/201 (45)	0.58 (0.35–0.95)	0.03			
Mean age, y (range)	50.55 (14–84)	49.78 (4–89)	1.00 (0.99–1.01)	0.62			
Previous hospitalization at ICU admission							
Previous stay in another unit during hospitalization	75/101 (74)	163/201 (81)	0.84 (0.44–1.60)	0.60			
Previous stay in the ED during hospitalization	62/103 (60)	125/201 (62)	1.07 (0.65–0.77)	0.78			
Length of ED stay, d	2 (0–55)	1 (0–37)	1.08 (1.01–1.15)	0.02		1.10 (1.02–1.19)	0.01
ED stay >2 d	34/103 (33)	35/201 (17)	2.45 (1.40–4.32)	0.002			
Days of hospitalization before surveillance culture, median (range)	3 (1–95)	2 (1–37)	0.99 (0.99–0.99)	<0.001			
Transfer from another hospital	43/101 (43)	51/193 (26)	2.79 (1.26–3.68)	0.005		2.52 (1.07–5.89)	0.03
Previous hospitalization	52/85 (61)	63/163 (38)	2.91 (1.53–5.52)	0.001			
Clinical characteristics at ICU admission							
Infection	63/101 (63)	82/140 (42)	2.62 (1.52–4.54)	0.001		1.76 (0.56–5.50)	0.33
Sepsis	46/62 (74)	54/81 (66)	1.41 (0.52–3.85)	0.50			
Surgery before ICU admission	53/102 (52)	106/194 (55)	0.92 (0.53–1.62)	0.78			
Trauma	8/100 (8)	25/194 (13)	0.62 (0.28–1.40)	0.25			
Stroke	5/100 (5)	17/194 (9)	0.61 (0.17–2.18)	0.45			
Severity scores							
SAPS 3, % median (range)	22 (4–92)	16 (0–98)	1.01 (1.002–1.02)	0.01		1.01 (1.002–1.03)	0.02
SOFA, median (range)	5 (0–19)	5 (0–19)	1.09 (0.95–1.07)	0.77			
Invasive procedures and devices							
Dialysis	14/100 (14)	11/194 (6)	2.50 (0.97–6.42)	0.06			
Tracheostomy	2/99 (2)	1/194 (0)	4.92 (0.36–44.67)	0.26			
Colostomy	2/99 (2)	2/194 (1)	2.00 (0.28–14.34)	0.49			
Upper digestive endoscopy	10/101 (10)	5/194 (3)	3.70 (1.11–12.32)	0.003		18.9 (1.83–195.98)	0.01
Colonoscopy	2/101 (2)	0/194 (0)					
Parenteral nutrition	2/101 (2)	1/ 194 (1)	3.77 (0.19–74.94)	0.38			
Underlying conditions							
CCI score, mean (range)	3.10 (0–9)	2.98 (0–11)	0.99 (0.96–1.02)	0.48			
Smoking	25/62 (40)	46/137 (34)	1.17 (0.49–2.78)	0.72			
Diabetes mellitus	20/102 (20)	44/198 (22)	0.86 (0.46–1.62)	0.65			
Malignant neoplasm	9/102 (9)	23/198 (12)	0.77 (0.35–1.70)	0.52			
Rheumatologic or autoimmune disease	11/102 (11)	16/198 (8)	1.44 (0.66–3.15)	0.36			
Cirrhosis	15/102 (15)	11/198 (5)	2.25 (0.85–5.91)	0.10			
Chronic kidney disease	12/102 (12)	14/198 (7)	1.51 (0.56–3.99)	0.40			
Solid organ transplant	8/102 (8)	16/198 (8)	0.62 (0.23–1.64)	0.33			
HIV infection	3/100 (3)	7/198 (4)	1.13 (0.27–4.76)	0.86			
Hematological malignancy	2/102 (2)	6/198 (3)	0.59 (0.13–2.87)	0.52			
Hematopoietic stem cell transplant	1/102 (1)	1/198 (0)	2.00 (0.12–32.42)	0.63			
Antimicrobial drug use							
Any drug at ICU admission†	81/99 (81)	142/193 (71)	1.56 (0.83–2.91)	0.161			
Carbapenem at ICU admission†	25/80 (31)	12/141 (9)	3.92 (1.51–10.21)	0.005		4.62 (1.30–16.40)	0.02
Any drug use in previous 3 mo	50/72 (69)	48/145 (33)	5.38 (2.31–12.53)	<0.001			

*Values are no. (%) except as indicated. CCI, Charlson Comorbidity Index; ED, emergency department; GCS, Glasgow Coma Scale; ICU, intensive care unit; OR, odds ratio; SAPS 3, Simplified Acute Physiology 3, presented as prediction of mortality risk in percentage; SOFA, Sequential Organ Failure Assessment. †Initiated >48 h before ICU admission.

**Table 2:** Multivariate analysis for potential factors associated with colonization by carbapenem-resistant *Enterobacteriaceae* at ICU admission, Hospital das Clínicas, São Paulo, Brazil, September 2015–July 2017*

Covariate	OR (95% CI)	p value
ED stay >2 d	5.85 (1.94–17.65)	0.002
Transfer from another hospital	2.10 (0.95–4.78)	0.076
SAPS 3 score	1.02 (1.003–1.03)	0.02
Carbapenem use on ICU admission, initiated >48 h before ICU admission	4.78 (1.31–17.47)	0.02
Infection at ICU admission	2.86 (1.08–7.55)	0.03
Upper digestive endoscopy	16.40 (2.16–124.50)	0.01

*Model using length of ED stay as dichotomous variable. OR, odds ratio; ED, emergency department; ICU, intensive care unit; SAPS 3, Simplified Acute Physiology Score III.

## Results

We included 304 patients in the study, 103 cases and 201 controls, and collected surveillance cultures for all patients. Of the 103 case-patients, 99 were colonized by *K. pneumoniae*, 2 by *Enterobacter cloacae,* and 2 by *Escherichia coli*. Of the 304 total patients, 188 patients (62%) were admitted to medical ICUs and 116 (38%) to surgical ICUs. Sixty-five patients were admitted directly to the ICU: 38 transferred from another hospital, 17 came from the operating room, and for 10 patients, this information was not available. Eighty-six patients were transferred from another ward and 152 from the ED; information was not available for 1 patient. Sixty percent of cases and controls stayed in the ED for some time during their hospitalization.

**Figure 2:**
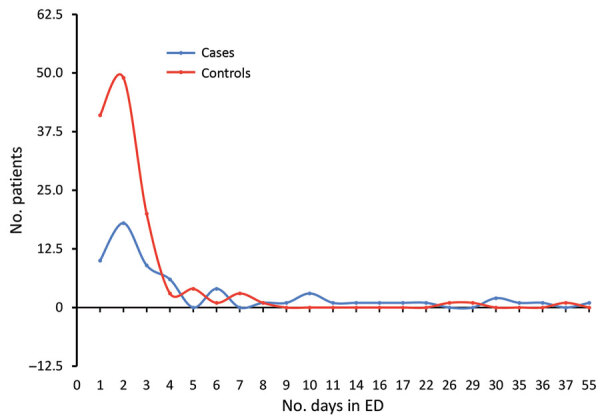
Distribution of days of stay in the emergency department (ED) comparing patients subsequently admitted to an intensive care unit who had a positive carbapenem-resistant *Enterobacteriaceae* culture within 2 days of admission (cases) and patients whose culture was negative (controls), Hospital das Clínicas, São Paulo, Brazil, September 2015–July 2017.

**Figure 3:**
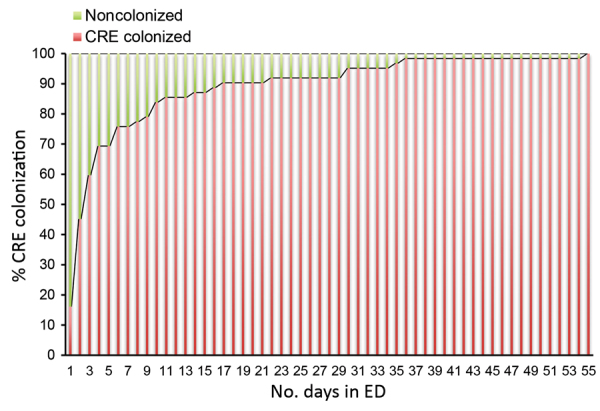
Distribution of colonization of CRE in patients admitted to an intensive care unit after a stay in the ED, Hospital das Clínicas, São Paulo, Brazil, September 2015–July 2017. CRE, carbapenem-resistant *Enterobacteriaceae*; ED, emergency department.

We performed bivariate analysis and demonstrated that 11 characteristics were associated with CRE colonization at ICU admission: sex, ED length of stay, ED stay >2 days, number of hospitalization days before the surveillance culture, transfer from another hospital, previous hospitalization, having an infection on ICU admission, clinical severity (SAPS 3 score), use of antimicrobial drugs in the previous 3 months, carbapenem use on ICU admission (initiated >48 hours before ICU admission), and upper digestive endoscopy (Table 1). The most common infections at ICU admission were pneumonia (37%), skin and soft tissue infection (14%), and CLABSI (10%). The median length of stay in the ED was longer for cases (2 days, range 0–55) than for controls (1 day, range 0–37; p = 0.02) (Figure 2). We analyzed the length of stay in the ED with the decision tree tool; we selected a stay >2 days as cutoff for this variable (χ^2^ = 12.799; p = 0.017). We found that 38/62 (61%) of the patients with CRE colonization at ICU admission were already colonized after 3 days of hospitalization in the ED (Figure 3).

We performed multivariate analysis with 2 models, using ED length of stay as a continuous or a dichotomous variable (>2 days). ED stay was a risk factor for colonization by CRE in both analyses: continuous (per day, odds ratio [OR] 1.10, 95% CI 1.02–1.19; p = 0.01) (Table 1) and >2 days of hospitalization (OR 5.85, 95% CI 1.94–17.65; p = 0.002) (Table 2). Use of carbapenem at ICU admission (initiated >48 hours before ICU admission), Simplified Acute Physiology Score III (SAPS 3), transfer from another hospital, and upper digestive endoscopy were risk factors for CRE colonization at ICU admission (Table 1).

Patients colonized by CRE at ICU admission had higher rates of infection by CRE (18 [18%]) than did patients not colonized by CRE when they sought care (11 [6%]; p = 0.001). Colonized patients also had higher in-hospital mortality rates (38 [38%] for patients colonized by CRE and 48 [24%] for those not colonized; p = 0.016).

## Discussion

Our results confirm our hypothesis that ED stay is a risk factor for CRE colonization in patients at the time of admission to the ICU. Other risk factors are use of carbapenem at time of ICU admission (carbapenem use initiated >48 hours before ICU admission), SAPS 3, upper digestive endoscopy, and transfer from another hospital (Table 1).

Including ED stay as a risk factor is a notable new finding. A stay in the ED is usually not considered to be a risk factor for CRE colonization (*9*). In a previous study, our group demonstrated that patients admitted to the ED had 6.8% prevalence of CRE colonization at admission to the ED and 18% acquisition rate for patients hospitalized in the ED for longer than 1 week. Six patients who were not treated in a healthcare facility were colonized by CRE at ED admission, implying circulation of this resistance mechanism in the community (*5*). Our findings show that ED hospitalization is indeed a risk factor for CRE colonization on ICU admission, whereas a previous stay in another hospital unit was not.

Although it is not common, CRE can be found outside the hospital. CRE has been described in community sources of water in Italy (*10*), Brazil (*11*), and Sweden (*12*); in chicken meat in Egypt (*13*); in vegetables imported from Asia (*14*), and in hospital sewage in Brazil, China, and Spain (*15*). Community-acquired CRE infection is difficult to determine; however, up to 30% of patients with CRE infection on hospital admission have had no previous exposure to the healthcare system.

The acquisition or transmission of CRE in the ED may be a result of the work overload. Ours is a tertiary-care public hospital in Brazil with an overcrowded ED. It is not unusual to have patients with high-complexity illness hospitalized on stretchers for longer than a week because of a shortage of ICU or ward beds to which to transfer patients or to have a low ratio of healthcare workers per patient. Prolonged ED stays probably facilitate cross-transmission of multidrug-resistant organisms such as CRE. Although on first thought the problem may be considered a local one, specific to our hospital and setting, this problem extends to other Brazil hospitals. Two other hospitals reported long stays in the ED, with 1 hospital reporting a median length of stay of 3 days (*16*) and another reporting that 21% of patients stayed in the ED for >5 days (*17*). Mortality rates in the EDs of these hospitals are high as well: 7.4% at the first and 3.9% at the second. Furthermore, we expect long ED stay is a problem in other countries, although seldom reported (*18**–**20*). Lack of access to healthcare in developing countries leads to other problems: healthcare-associated infection rates are much higher in developing countries than in high-income countries (*21*), as are drug resistance rates (*22*). In a disadvantaged healthcare system, patients with known risk factors (*23**–**25*) are often hospitalized for prolonged periods in the ED and are a potential source of multidrug-resistant bacteria for other patients in the ED and ICUs.

The need to establish strategies to control CRE transmission in EDs and hospitals is urgent; resistance is not an isolated problem in a specific hospital unit or even in a specific hospital. High workload, understaffing, and turnover of healthcare workers make it difficult to improve adherence to hand hygiene in the ED; additional strategies are needed (*26*,*27*), and interventions must be multimodal. These interventions must include a change in the workflow of the ED and hospital as well as the entire health system to reduce overcrowding (*26**–**28*). The lack of infrastructure in the ED puts patients in stretchers too near to each other, probably facilitating cross-transmission. In this scenario, good hand hygiene may not be achievable. Dividing patients into cohorts and assigning dedicated staff may reduce transmission of CRE (*29*). Hospital staff should discuss screening strategies for CRE and early isolation and contact precautions in the ED (*30*). Rising antimicrobial resistance is a substantial threat to global health (*31*), and prolonged ED hospitalization may play a major role in hospital-acquired resistance in low- and middle-income countries.

We found other risk factors that have already been associated with CRE colonization, including transfer from another hospital (*24*,*25*), use of carbapenem (*23**–**25*), SAPS 3, and upper digestive endoscopy (*32*). All of them are associated with previous exposure to healthcare or severity of patients (*33*). The previous use of carbapenems is well described as a risk factor for CRE colonization (*23**–**25*,*33*). In our study, the patients were using carbapenem for ≥48 hours by the time of surveillance culture. Although this timeframe is short, it may have been sufficient for selection of carbapenem-resistant bacteria. We must emphasize that, even though carbapenem use was an independent risk factor in multivariate analysis, the attending physicians may have prescribed it because after a certain length of time in the ED, the patient is at risk for infection by antimicrobial-resistant bacteria.

Of interest, although cirrhosis was not associated with CRE colonization, upper digestive endoscopy was, which suggests that the risk for colonization after endoscopy is probably due to the procedure itself and not to the patient’s underlying conditions. We found no clusters of endoscopy-related CRE colonization in the study period, suggesting that it was not an outbreak. Colonization may be a result of improper cleaning procedures. Because this was a retrospective study, we could not test the endoscopes for CRE colonization at the time that colonization occurred. Prospective surveillance for endoscopy-related CRE is underway.

It is difficult to assess the influence of local factors in the hospital ED on colonization by CRE. Factors such as low adherence to hand hygiene and contact precautions, proximity of beds, and others work together to facilitate the transmission of microorganisms. A limitation of this study is that it was not possible to evaluate the effect of each of these variables individually. Other limitations of our study were the retrospective nature of a case–control study; missing data for some variables; potential bias of retrospectively obtaining data from medical records; and the fact that the study was done in only 1 hospital, requiring confirmation in other centers or a multicenter study.

In conclusion, this study demonstrates that prolonged ED stay is a risk factor for CRE colonization at the time of admission to the ICU. Other risk factors were the use of carbapenems at ICU admission (initiated <48 hours before ICU admission), SAPS 3, upper digestive endoscopy, and transfer from another hospital. Clinicians should be aware of the implications of these findings and implement interventions in the ED to control CRE in other hospital units.
